# Metallic Dies

**Published:** 1856-07

**Authors:** P. H. Austen


### ARTICLE IV.

### *Metallic Dies.* By Professor P. H. Austen.

*(Continued from July No., 1855.)

The table on page 367 is designed to present in one view the relative value of certain metals and metallic alloys, in respect of the three properties—fusibility, hardness, contractility. In expressing the composition of the alloys the initials of the English names are used, not as in chemical works, of the Latin names. This is done because to the majority of readers, the initials A. L. T. will more instantly suggest the names, antimony, lead, tin, than the corresponding Latin initials, Sb. Pb. Sn.

The scale of fusibility is prepared in part from reliable sources, and in part from direct experiment. The scale is expressed in round numbers. In the case of lead, one authority gives 594°, another 612°; 600° is nearly the mean between the two. In the case of zinc, 773° is the fusing point according to Daniell, and 1996° that of copper. In the first place, it is very doubtful whether these high temperatures can be ascertained exactly to a degree; in the second place, the design of this table does not require such exactness.

The scale of hardness has been carefully prepared in the manner described on page 373*. A hammer of 7 lbs. weight, falling from a height of nine inches upon a steel punch 1-6th inch in diameter, indented the several samples of metals, ¼ inch thick, placed on a heavy anvil. The depth of the indentation is expressed in thousandths of an inch, and is in each case the average of from 6 to 12 trials. For example, in nine samples of zinc, the punch sank to the depths respectively 17, 20, 17, 17, 18, 18, 20, 18, 18, a difference so slight as to confirm rather than invalidate the accuracy of the experiments.

The micrometer screw used in these experiments was made by Mr. John Jones of this city, and works with most beautiful accuracy. The screw has 40 threads (square cut) to the inch, and carries on its head an index which moves over a circle graduated into 25 parts. These divisions, being about ¼ of an inch apart, are easily read, and register each, 1000th part of an inch. The readers of the Journal will obtain a correct idea of these minute divisions of an inch from the following statement. Each leaf of the Journal is 5-1000th of an inch thick; 10 leaves, (20 pages,) 5-100ths; the thinnest letter paper, about the 1-1000th. The shrinkage of an average sized zinc die from outside to outside of the alveolar ridge, measuring two inches, will be 27-1000ths, being on each side a space nearly equal to the thickness of three Journal leaves. From inside to inside of the lower jaw, we have about the same average. In the first case, the plate would "bind," and if the ridge were covered by an unyielding mucous membrane, it would prevent accuracy of adaptation. In the second case, the plate would have too much lateral "play," and consequently lack stability. Again, in a moderately deep arch, say a half inch in depth, the shrinkage between the level of the

* The references in this number are to the July No., 1855.

ridge and the floor of the palate will be nearly 7-1000th—rather more than one leaf of the Journal. In the deepest arches this shrinkage becomes a serious difficulty; in the shallower cases, it is not of much moment, as there is no mouth so hard as not to yield the 1 or 2-1000ths of an inch.

It is scarcely necessary to remark, that the softer the metal the higher its number in this scale. In the columns of contractility and fusibility, the lower numbers represent those metals which are most fusible and have least shrinkage. The metals and alloys enumerated have a very different value in the three scales; of course the intrinsic value of any one must be determined by the relative importance of these three properties, in consideration of the purpose to which it is to be applied.

The scale of hardness has been prepared with much care, because it is the first, to the writer's knowledge, ever attempted. We find, as intimated on page 373, in most works on metals, a "Scale of Hardness," but this indicates not resistance of the metal under a blow, but merely the comparative ease with which it may be scratched. In this scale, the six metals stand in the following order: copper, bismuth, cadmium, tin, antimony, zinc, lead; an order that does not indicate correctly their relative value to the dentist.

Cadmium, antimony and bismuth do not appear in the table. The first, because of its scarcity and costliness, and the two last, because of their extreme brittleness, are not adapted to practical use. Antimony and bismuth both rank lower in the scale of hardness than zinc, but they were too brittle to stand the blow of the hammer, and no indentation could be obtained by measurement of which they could be compared with any accuracy. Lead is in reality softer than its number 138 would indicate. Experiments with an ingot ½ inch thick, place it as high as 144, being exactly 8 times softer than zinc. This is the only case in which the increased thickness of the ingot affected the result.

The scale of contractility is expressed in decimal fractions of an inch, and indicates the amount of contraction, per linear inch, that takes place in each metal between its point of fusion and a temperature of 74°. The scale is made by careful measurements of ingots 3 inches long, $\frac{5}{8}$ inch wide and $\frac{1}{4}$ inch thick, poured into a soap-stone ingot mould. The ingot mould of soap-stone has the two fold advantage of retaining its heat, so as not to allow the metal to chill too suddenly, and of not itself expanding by heat. A metallic ingot mould would, by its own expansion, either destroy the accuracy of the experiments, or at least would greatly complicate the process. The writer proposes in the repetition and extension of these experiments, to use plumbago instead of soap-stone, as being possibly more entirely free from any expansion. The scale here given may, however, be relied upon as sufficiently accurate to guide in the choice of metals.

In Ure's Dictionary, and elsewhere, may be found tables giving the linear expansion of metals between 32° and 212° Assuming the ratio of expansion to be uniform up to the melting point, it is easy to find the total expansion (or contraction) of each metal. We should thus have the total contraction for zinc, .01162; for lead, .00858; for tin, .00495. The results by calculation are, in all three cases, a little less than those deduced from experiment—a discrepancy arising from the fact established by Lavoisier and Laplace, that solids as well as liquids expand in an increasing, not in a uniform, ratio.

From the various tables alluded to, we find the average contraction of the 10 following metals and alloys, between the boiling and the freezing points of water, to be for

| | | | |
|---|---|---|---|
| Platinum | .00091 | Gun metal | .00182 |
| Antimony | .00108 | Brass | .00191 |
| Cast iron | .00111 | Tin | .00236 |
| Bismuth | .00139 | Lead | .00285 |
| Copper | .00180 | Zinc | .00298 |

An examination of this table, and comparison with the following one, give us some interesting facts.

On page 380, (July, 1855,) a very common error with respect to the effect of antimony in type metal was noticed. We here see, not that antimony expands under decrease of heat, but that its rate of contraction is to that of lead nearly as 1 to 3, and that, when added to lead in the proportion of 1 to 5, it diminishes the total shrinkage nearly one half. Antimony, iron, bismuth and zinc may all, in the moment of passing from the liquid to the solid form, by virtue of their crystalline tendency, slightly expand, and so fill the fine lines of a delicate matrix; but once solid, they obey the universal law, and contract under decrease of temperature.

Again we see verified the statement made on page 375. The actual shrinkage of a metal is dependent not only upon its ratio of contraction, but also upon its point of fusion. Tin, with a rate of contractility more than twice that of iron, has not half the actual shrinkage. Zinc contracts more rapidly than copper or brass; yet, starting from a lower temperature, its total shrinkage is much less.

| | Melting Point. | Contractility. | Hardness. | Brittleness. |
|---|---|---|---|---|
| 1 Copper | 2000° | .02236 | .020 | 1 |
| 2 Brass 2 C—1 Z | 1870° | .02216 | .024 | 2 |
| 3 Zinc | 770° | .01366 | .018 | 5 |
| 4 1292 Z—579 tin | 660° | .01233 | .024 | 4 |
| 5 Lead | 600° | .01066 | .138 | 1 |
| 6 5 type metal 5 L—1 A | 500° | .00633 | .045 | 10 |
| 7 Tin | 440° | .00633 | .054 | 1 |
| 8 2 L—1 T | 440° | .00633 | .050 | 3 |
| 9 1 L—2 T | 340° | .00500 | .040 | 3 |
| 10 2 L—3 T—1 A | 420° | .00433 | .026 | 7 |
| 11 5 L—6 T—1 A | 320° | .00566 | .035 | 6 |
| 12 5 L—6 T—1 A—3 B | 300° | .00266 | .030 | 9 |
| 13 1 L—1 T—1 B | 250° | .00066 | .042 | 7 |
| 14 1 L—1 T—1 B | 250° | .00066 | .035 | 6 |
| 15 5 L—3 T—8 B | 200° | .00200 | .045 | 8 |
| 16 2 L—1 T—3 B | 200° | .00133 | .048 | 7 |

The last column contains an approximate estimate of the relative brittleness of the 16 samples given. As in the other columns, the low numbers represent the metals, so far as this property is concerned, most desirable. Those marked below 5 are malleable metals; those above 5 are brittle; zinc, marked 5, separates these two classes and belongs to one or the other, according to the way in which it is managed. Melted and poured into a shallow mould, it may be broken. Even in the more compact form of a well shaped die it is possible by undue violence to crack it; an accident the liability to which is necessarily increased, when the die is made too shallow or too flaring. Meeting with some such vexatious accident, one might well ask, taking in hand a piece of sheet zinc and bending it in any direction, if these two can be one and the same metal. The only difference is that sheet zinc is annealed zinc. By annealing zinc, we take it out of the class of brittle metals, and fit it for the malpractice of the most unskillful operator; not meaning to say that the most skillful may not at times gladly avail himself of the change. In the manufacture of sheet zinc it is rolled at a temperature between 212° and 300°, retaining its malleability when cold. This change from the brittle to the malleable state takes place at about 230°; continues unchanged up to 300°. After this it becomes less malleable, and at 450°, or the melting point of tin, it becomes again brittle. The simplest way to anneal a zinc die is to place it in the melting ladle with about a table-spoonful of water, removing it in 30 seconds after the water has boiled away. If the fire is a very hot one, remove it immediately on the disappearance of the water. It will often happen that the die is annealed in the process of taking the counter-die. This will more certainly occur when *Nos. 7, 8 or 9 are used for the counter. For example, take tin: using a mass twice the size of the die, should it be heated to 540°, (100° above melting point,) it would not, allowing for loss of heat by radiation and contact with the cast iron ring, (if one be used,) heat the zinc beyond 330°. Lead, cast as cool as it could possibly be poured, unless in a very heavy ring (such as a "cart wheel box") or in quantity too small for a well shaped counter, would be apt to raise

* When metals are named thus by numbers, reference is had to the table on page 367.

the zinc at least to 400°, and so impair its malleability, whilst, if poured as hot as many are in the habit of doing, the zinc will remain as brittle as when first cast.

The other brittle metals and alloys may be rendered somewhat less brittle by immersion in boiling water, except the two last. In the course of the experiments necessary to the preparation of this report, a number of alloys, valuable in respect of fusibility, contractility and hardness, were found useless because brittle. Further experiment may enable us to assign to some of these the requisite toughness. Meanwhile it is hoped that the table here given may induce others to prosecute inquiries for themselves.

The design of this report being rather to offer practical suggestions than to indulge in theoretical refinements, I have taken the metals as they are found in commerce. The copper of commerce almost always contains traces of arsenic, nickel and tin, which impair its malleability. Commercial zinc always contains iron, lead, copper, cadmium. Block tin generally contains antimony, copper or lead. Lead is usually nearly pure. Of course these impurities affect more or less the properties of the metals; but practically they may be disregarded except when, as in No. 4, we wish to make an alloy of very exact proportions. This is a mixture of 40 atoms of zinc to 10 of tin, and is the only instance in the table of an attempt to unite two metals in their atomic proportions. Theoretically such mixtures are the most perfect; but practically there is a two-fold difficulty; first, a want of absolute purity of the metals which necessarily destroys the accuracy of the proportions; secondly, the impossibility of calculating with absolute certainty the loss by oxydation of the more fusible component of the alloy. Hence, in the composition of the brasses, bronzes and pewters it is usual to make such additions of the separate metals to a lot of old alloy, as the judgment and experience of the founder may dictate, due allowance being made for the burning out of the more fusible metal.

The slight commercial impurities of copper, zinc, lead and tin may, in the making of metallic dies, be safely disregarded by the dentist. A much more important point is the necessity of using clean melting ladles, and of having more than one. Take in illustration a very common case. Zinc is melted in a ladle containing the remnant of a previous melting of lead. The lead, so much the heavier, remains at the bottom of the ladle, and, if *all* the metal in the ladle be poured, sinks to the bottom of the mould. The die is taken from the sand when the zinc has set, and the more fusible lead runs out, leaving the die imperfect on its face: or if allowed to cool entirely, it will, while apparently perfect, have a part of its alveolar ridge composed of lead; consequently too soft to swage the plate. This accident may be avoided in either of three ways: use separate ladles; or avoid pouring all the metal, which leaves the heavier lead in the ladle; or use tin for the counter-die. In the last case the zinc readily alloys with any remnant of tin, having very nearly the same specific gravity, and the only result, from repeated meltings, will be a softening of the zinc in proportion to the tin added.

In the use of the more fusible alloys of the table, from type metal down, a copper ladle and spirit lamp will suffice for their fusion. In fact, flame heat will be better than an anthracite or charcoal fire, from the less liability of overheating. Excess of heat wastes the more oxydizable metal and alters the proportion of the alloy; and where a copper ladle is used, a coal fire will very quickly raise the metal to a temperature, at which it will alloy itself with the copper, destroying the ladle and letting the metal quietly down into the fire. There is a tinned iron (hammered, not sheet-iron) ladle of French manufacture, very convenient in size, very durable, and not liable to this last accident attending the careless use of copper. In using either the tinned iron or copper, the ladle should after each melting be instantly scraped with a spatula or wiped out with paper or shavings: otherwise the proportions of the alloys, where several kinds are used, will become changed.

Most of our readers may prefer to draw their own inferences from the table given, and select such metals or alloys as may best suit their particular views, convenience or custom. And the scope of this article would be greatly mistaken if we should be understood as wishing otherwise, or as dictating to any one a method or material suitable for all cases. A few suggestions, however, may not be amiss. Our concluding observations upon the properties of metals will be more conveniently made by taking them seriatim, as given in the table, interposing some brief notice of metals and alloys not found there.

Iron.—Fuses at 2786°, sp. gravity 7.78. Cast iron ranks as a brittle metal, but in the solid form of a die would resist any necessary force. Having a surface hardness almost equal to steel, it would admirably suit the dentist, but for two serious objections, its high fusion point and its consequent great contractility: we might also add its roughness of surface, which in so hard a metal would indent the plate. As a component of alloys, it is used in small quantity to harden copper, and most probably the zinc of commerce may owe a slight increase of hardness to the trace of iron it contains.

Nickel.—Never used in the arts in its pure state. Less fusible than iron, it diminishes the fusibility of the alloys of which it is a component. It is chiefly used in the manufacture of German silver, albata, the *white silver* of the Chinese, &c., all of which are too infusible, contractile and costly to be used for metallic dies. We have made no attempt to alloy it with the more fusible metals.

Copper, Brass, Bronze.—The two first are shown in the table to be inferior to zinc in hardness, decidedly inferior in fusibility, and much more contractile. Zinc, superior in all except its brittleness, when made tough by annealing, is therefore unquestionably preferable to either. For the purpose now under consideration we should entirely exclude from the laboratory, copper, or any of its various alloys with zinc, forming brass, Bath metal, sheathing metal, pinchbeck or mosaic gold. The hardness of zinc will be increased slightly by the addition of 30 or 50 per cent. of copper, but it increases also the shrinkage, and makes it harder to melt.

The alloys of copper with tin, forming bronze, gun metal, bell metal, speculum metal, &c., have the advantage over those with zinc of being very much harder. They are all, however, more brittle, and are all as objectionable on the score of infusibility and shrinkage. To those wishing to make trial of bronze we would recommend alloys of 6, 8, 9, 10 and 12 copper to 1 tin. The first is most brittle, the last is softest. In melting, care must be taken to prevent the waste of the tin, which readily takes place at the high temperature of melted copper. The purest "grain" tin and Swedish copper should be used in making bronze. 2 tin and 1 copper, form what is called "temper," and may, perhaps, be advantageously employed in hardening tin, using 1 part temper to 100 parts tin. The formulæ given in the table for hardening tin are, we think, better, because more readily fused.

The alloys of copper and tin, if not very useful to the dentist, are interesting as illustrative of the remarkable results that so often, in the alloying of metals, defy all attempts at *a priori* reasoning. There are some general rules that may measurably guide in originating or in modifying alloys—the physical properties of the components, entering, as it were, into mutual compromise. Thus we expect to find the alloy of 2 brittle metals itself brittle—so it always is. A brittle metal, if in equal parts, will usually render a tough one brittle: if either be in excess, the result will vary accordingly—the brittle metal, however, having always the more influence.

Alloys of two ductile metals defy all calculation as to their probable properties. Copper is highly ductile, and tin is very malleable; while speculum metal (2 C—1 T) is the most brittle of all alloys. Gum metal (9 C—1 T) is tough and rigid, neither malleable nor ductile: by adding tin, the *softer* of the two metals, the alloy is actually hardened, until, in the proportion of speculum metal, it cannot be cut with steel tools. Again, 6 C—1 T will not stand a heavy blow, yet its cohesive strength is twice as great as either component: and 1 bismuth, 4 tin has a cohesive strength 5 times greater than bismuth and 3 times greater than tin. In the case of ductile metals, where mixed in nearly equal parts, about half the alloys will be ductile, and half will be brittle; where either metal greatly predominates, the alloy is usually ductile: but the ductility of an alloy is seldom, if ever, equal to that of the more ductile constituents.

The influence of alloying upon fusibility is also remarkable. The alloy is invariably more fusible than the most refractory component, and often than the less refractory, provided the latter does not exist in too small quantity. Silver solder is a familiar illustration; copper less fusible than silver, makes the silver itself more fusible. So, again, the soft solders, except where the lead is in excess, are more easily melted than tin. But only actual experiment could have revealed the remarkable fact that certain alloys of lead, tin and bismuth, fusing respectively at 600°—440° 500°, will melt in boiling water.

Zinc.—This metal in its simple unalloyed state well deserves the important place it holds in the laboratory of the dentist, who moulds in sand. Metals or alloys harder than zinc are more infusible and liable to greater shrinkage: whilst those which are more fusible and contract less, gain these advantages at the expense of hardness. In the first class we have gun-metal, or bell-metal, and alloys of zinc with small proportions of iron, antimony and copper. In the second class may be instanced alloy No. 4 of the table, in which case we see that the hardness of the alloy is diminished in a greater ratio than its shrinkage, making it, by consequence, less valuable, the difference in the melting point of the two not being sufficient to give any practical advantage.

We have alluded elsewhere to the amount of shrinkage of zinc and the manner in which it prevents accuracy of adaptation. Summing up briefly, in the worst possible case—a very deep arch and the entire mucous surface *very* hard—*first*, the plate will bind on the outside and so not fit the ridge: but supposing it to fit down upon the alveolar border, it will *secondly* fail to touch the abrupt sides of the deep arch, and *thirdly* it will, by a still wider interval, fail to come in contact with the roof of the mouth. Many use zinc in such cases, burnishing or hammering the plate down upon the plaster cast; thus injuring the cast and only partially correcting the difficulty. Others resort to the practice of bending the edges of the plate. To those who adopt this expedient (without which we fear dental mechanical practice with many would soon to be at an end) we would suggest the propriety of dispensing with the air-chamber, so universally (and often so unnecessarily) used; inasmuch as by this method of bending down the edges of a plate touching in no other portion of its palatine surface they secure the largest kind of an air cavity.

A better practice than either of the above named is to complete the swaging upon a fusible metal (Nos. 11 to 16) die, with the same counter-die used for the zinc, placing pieces of paper or sheet lead upon the counter where the plate fails to fit the new die. By this means we avail ourselves of the hardness of zinc and the non-contractility of the fusible alloy. In the writer's judgment this is the best plan in all cases where zinc alone will not give a secure adaptation. It may be well here to caution the operator who is inexperienced in the use of fusible alloys. When poured into a sand matrix, they retain their fluidity much longer than zinc, and are very liable to be spoiled by the upward escape of the vapor of the sand. The sand mould should be thoroughly dried, and then allowed to cool. This practice of drying the mould is safest in every case, whether with zinc or fusible metal. If the haste of the operator will not permit this, it may be well for him to know that by dampening the sand with *soapy water he will materially lessen the risk of "blowing." Why soap should exercise any such influence we must leave to others, or to another opportunity, to inquire.

Lead.—In its pure state is useful only as a counter-die; for which purpose it is improved by the addition of antimony, forming various grades of type metal; or of tin forming soft solder, or pewter. The composition of type metal varies in different foundries, and is usually kept secret. There is often a small per centage of copper and tin and sometimes of bismuth; but the chief ingredient is antimony, varying from $\frac{1}{4}$ to $\frac{1}{8}$. The former is used for the smallest types, is hard and very brittle; the latter is used for the largest types. An alloy of medium sized type and lead, equal parts, forms an excellent material for the counter to a zinc die: so also is alloy No. 8, and either is cheaper than tin alone.

Tin.—In its pure state, more suitable for the counter than for the die, though used for this latter purpose by many who practice the "dipping" method. To such we would recommend, for the counter die into which the cast is to be dipped, lead 9, antimony 1, or lead 4, tin 1; or pure tin, and for the die, alloy No. 9, or some of the more fusible alloys, Nos. 11 to 16. In this selection, it is to be remembered that the metal for the die must be the harder and at the same time the more fusible, whilst both should shrink as little as possible. It is commonly thought that the shrinkage of the counter compensates that of the die, and makes the latter a fac simile of the plaster cast. A few moments' reflection, with the aid of a simple sectional diagram, will convince any one of this error, bearing in mind that all shrinkage is to be measured towards the centre of the mass.

* For this hint we are indebted to Dr. R. T. Reynolds, of Philadelphia; and recently we had brought to our notice an instance in which a locomotive was stopped from inability to generate steam because one of the men had washed his hands with soap in the water used in the boiler. The boiler had to be entirely emptied and re-filled before a head of steam could be raised.

Those practicing this method of obtaining dies, cannot be too strongly impressed with the importance of keeping the two kinds of metal as distinct as possible, and so marked that they will not be mistaken each for the other. The greater care is necessary when the alloys resemble each other. It would be a vexatious mistake to dip the cast in No. 9 and pour No. 8 upon it; quite as annoying this, as to have lead in the zinc ladle; or, where die and counter are made of the same metal, to pour the latter too hot. The first accident requires for its avoidance systematic order in the laboratory, while ordinary care will prevent the two latter. The slovenly or confirmedly careless operator had better avoid having a variety of metals or alloys, as his frequent mistakes will more than balance any advantage to be derived from their use.

We shall conclude this article, which has already exceeded its intended limit, by a few brief remarks upon the more fusible alloys of the table. Bismuth is an essential component of all such alloys, and seems to have greatest effect upon their fusibility, although itself more infusible than tin. For all ordinary purposes, No. 13 will answer. No. 14 differs only in having been repeatedly used, thereby being more thoroughly mixed. It becomes closer grained, is harder and less brittle. Where a still more fusible metal is desired, the proportion of bismuth should double. In Nos. 15 and 16, the weight of bismuth is equal to that of the tin and lead together. So in Rose's fusible metal, (L. 1, T. 1, B. 2,) and an alloy 3 L., 2 T., 5 B., said to fuse at 199°.

The difference in these four last named is in the varying relative proportions of lead and tin. They fuse at nearly the same temperature, but vary in their degree of brittleness. Further experiment is required to determine which of all possible combinations of these three metals is best. If bismuth is in larger proportion, the alloy will be too brittle. The same will happen if the tin exceeds the lead The alloys No. 15, 16, will, we think, be found to answer every purpose required of this class.

These metals must be repeatedly melted to insure their thorough intermingling. The union is probably in part chemical and part mechanical. To this partial mechanical mixture is perhaps to be attributed the pasty, mush-like consistence of these alloys in their semi-fused state—the more fusible component melting and holding in suspension the particles of the less fusible ingredients. In this state it may be taken up in a spoon and dashed on to a green plaster cast, which should be cold and unvarnished, and may be surrounded merely with a strip of paper. In a few moments it will be hard enough to keep its shape without the paper, which may then be removed and the sides of the cast trimmed to any required shape. At a temperature of about 150° to 175° it can be cut like cheese. Another method of obtaining the counter die, is by the *clichee* process. Pour the metal into a suitable ring, remove the film of oxyd from the surface with the edge of a card, then when the metal is just on the point of setting, bring the cast down upon it with a quick steady motion. If properly done, the impression will be very sharply defined and free from any air holes; but to do it skillfully requires practice. The counter-die obtained by either process must be allowed to cool in the air, not in water, for, if there is the slightest trace of dampness on the surface, it will spoil the die next to be taken. Adhesion of the die and counter where the same metal is used for both, is prevented by, 1st, coating the counter with whiting, 2d, having it quite cool, 3d, pouring on the metal for the die in a pasty, semi-fluid condition.

Some of our readers may think that on some points we have been unnecessarily plain and diffuse; others may detect errors of statement; and others wish that we had been more specific and explanatory. The first should remember that what is to them an old story may to others be quite new: to the rest we repeat the remark with which we began our essay, that it is not claimed to be either infallible or exhaustive. Our object will have been fully answered if we shall have suggested to some any new and useful idea, or shall incite others to the further investigation of a subject that we feel convinced will repay a more extended research.

